# Editorial: Methods in radiation oncology

**DOI:** 10.3389/fonc.2024.1388519

**Published:** 2024-03-13

**Authors:** Amirhosein Kefayat, Mahshid Bahrami, Mojtaba Karami, Fatemeh Ghahremani

**Affiliations:** ^1^ Department of Oncology, Isfahan University of Medical Sciences, Isfahan, Iran; ^2^ Department of Radiology, Isfahan University of Medical Sciences, Isfahan, Iran; ^3^ Department of Dermatology, Tehran University of Medical Sciences, Tehran, Iran; ^4^ Department of Medical Physics and Radiotherapy, School of Paramedicine, Arak University of Medical Sciences, Arak, Iran

**Keywords:** cancer, radiotherapy, radiation oncology, metastasis, radiation - adverse effects

In recent years, radiotherapy has played a critical role for cancer patients’ treatment. Recently different radiotherapy methods have emerged and different studies have tried to maximize their efficacy while limiting their disadvantage (1). In this Research Topic editorial letter, we tried to address the most recent methods of radiotherapy, how they can conquer current challenges in different cancer radiation therapy, their therapeutic efficacy, and probable weakness.

Radiotherapy always has been a well-known treatment options for esophageal cancer patients who are not candidates for surgery. Two methods of radiation therapy including, involved field irradiation (IFI) and elective nodal irradiation (ENI) has been widely investigated for esophageal cancer. elective lymph node irradiation (ENI) aim is to irradiate to an area that has not been metastasized to decrease chance of metastasis formation which can improve local area control but did not improve overall survival (OS). On the other hand, ENI can increase risk of treatment-related adverse events. IFI irradiates only the affected area to limit the area of irradiation. According to the metanalysis published in our Research Topic (2), IFI offers significant improvements in overall survival rates at 5 years compared to ENI. While, it is associated with a notable decrease in the incidence of grade ≥2 acute esophagitis and grade ≥3 acute esophagitis, highlighting its potential to reduce treatment-related adverse events. The findings introduce IFI as a promising treatment method for esophageal cancer patients undergoing definitive radiotherapy or chemoradiotherapy.

Stereotactic body radiotherapy (SBRT) has been employed as a deeply effective treatment modality for lung cancer which not only can deliver precise radiation dose to the tumor site but also offer minimal damage to surrounding healthy tissues (3). However, intrafraction motion during SBRT can lead to target dose discrepancies, compromising treatment efficacy. Therefore, many research studies have focused on investigating the impact of intrafraction variation on target dose distribution in lung SBRT. The main issue in the lung SBRT is respiratory-induced target motion can lead to tumor geometric uncertainty and consequent reduction in the local control and increase the chance of off-target radiation delivery to nearby organs. Thus, respiratory motion must be managed and controlled during treatment. In this Research Topic one of the evaluated radiation therapy methods is SBRT for lung and we underscored the importance of accounting for motion effects in SBRT treatment planning to ensure optimal dose delivery and treatment outcomes.

This motion issue during fractions of radiation therapy has been mentioned for different tumors especially in the respiratory and gastrointestinal system while knowledge methods have introduced to solve this problem. Accurate monitoring of intrafraction tumor motion is an option for ensuring precise radiation dose delivery during radiotherapy and recent studies have investigated the accuracy of ultrasound tracking for monitoring tumor motion in real-time in pancreas tumor. Researcher demonstrated that ultrasound tracking exhibits high accuracy in tracking tumor motion and ultrasound tracking may represent a potential method for real-time monitoring of targets during radiotherapy, facilitating more accurate delivery of radiation doses and improving treatment outcomes for patients with pancreatic cancer and other tumors which move during irradiation and ultrasonography is possible for their traction (4).

Proton pencil beam scanning (PBS) transmission has gained a lot of interest as a potential treatment modality for lung cancer as it exhibits high toxicity-sparing effects and improved treatment outcomes. Plan quality and robustness of proton PBS transmission FLASH delivery in lung cancer treatment has been a hot topic so far for research studies. In this Research Topic we found out that while transmission plans have yielded slightly inferior plan quality compared to conventional proton SBRT plans, they have demonstrated improved robustness and potential for toxicity-sparing effects. By employing more beams, both dose and dose-rate robustness for transmission plans can be achieved, offering promising prospects for enhancing treatment efficacy and minimizing treatment-related adverse events in lung cancer patients (5).

In conclusion, the evolution of radiotherapy in recent years has been instrumental in advancing cancer treatment options. As highlighted in this editorial letter, various radiotherapy methods have emerged, each with its unique strengths and challenges. From the promising outcomes of IFI in esophageal cancer to the challenges of managing intrafraction motion in SBRT for lung cancer, researchers continue to innovate and refine treatment strategies. Additionally, the potential of ultrasound tracking and proton pencil beam scanning (PBS) transmission offers further avenues for improving treatment outcomes and minimizing adverse events. Overall, these advancements underscore the ongoing commitment to enhancing radiotherapy efficacy while prioritizing patient care and safety.

## Author contributions

AK: Conceptualization, Data curation, Formal analysis, Funding acquisition, Investigation, Methodology, Project administration, Resources, Software, Supervision, Validation, Visualization, Writing – original draft, Writing – review & editing. MB: Conceptualization, Data curation, Formal analysis, Funding acquisition, Investigation, Methodology, Project administration, Resources, Software, Supervision, Validation, Visualization, Writing – original draft, Writing – review & editing. MK: Conceptualization, Data curation, Formal analysis, Funding acquisition, Investigation, Methodology, Project administration, Resources, Software, Supervision, Validation, Visualization, Writing – original draft, Writing – review & editing. FG: Conceptualization, Data curation, Formal analysis, Funding acquisition, Investigation, Methodology, Project administration, Resources, Software, Supervision, Validation, Visualization, Writing – original draft, Writing – review & editing.
